# Oncolytic virotherapy in hepato‐bilio‐pancreatic cancer: The key to breaking the log jam?

**DOI:** 10.1002/cam4.2949

**Published:** 2020-03-04

**Authors:** Yuwei Li, Yinan Shen, Ronghua Zhao, Ismael Samudio, William Jia, Xueli Bai, Tingbo Liang

**Affiliations:** ^1^ Department of Hepatobiliary and Pancreatic Surgery The First Affiliated Hospital Zhejiang University School of Medicine Hangzhou China; ^2^ Zhejiang Provincial Key Laboratory of Pancreatic Disease Hangzhou China; ^3^ Innovation Center for the study of Pancreatic Diseases Hangzhou China; ^4^ Virogin Biotech Canada Ltd Vancouver Canada

**Keywords:** biliary tract cancer, hepatocellular carcinoma, immunotherapy, oncolytic virus, pancreatic cancer

## Abstract

Traditional therapies have limited efficacy in hepatocellular carcinoma, pancreatic cancer, and biliary tract cancer, especially for advanced and refractory cancers. Through a deeper understanding of antitumor immunity and the tumor microenvironment, novel immunotherapies are becoming available for cancer treatment. Oncolytic virus (OV) therapy is an emerging type of immunotherapy that has demonstrated effective antitumor efficacy in many preclinical studies and clinical studies. Thus, it may represent a potential feasible treatment for hard to treat gastrointestinal (GI) tumors. Here, we summarize the research progress of OV therapy for the treatment of hepato‐bilio‐pancreatic cancers. In general, most OV therapies exhibits potent, specific oncolysis both in cell lines in vitro and the animal models in vivo. Currently, several clinical trials have suggested that OV therapy may also be effective in patients with refractory hepato‐bilio‐pancreatic cancer. Multiple strategies such as introducing immunostimulatory genes, modifying virus capsid and combining various other therapeutic modalities have been shown enhanced specific oncolysis and synergistic anti‐cancer immune stimulation. Combining OV with other antitumor therapies may become a more effective strategy than using virus alone. Nevertheless, more studies are needed to better understand the mechanisms underlying the therapeutic effects of OV, and to design appropriate dosing and combination strategies.

## Graphical abstract

In this work, we systematically review and summarize the current status of treatment and the application of oncolytic viruses for HCC, pancreatic cancer, and biliary tract cancer. Most OV therapies exhibits potent, specific oncolysis both in cell lines in vitro and the animal models in vivo and in patients. What's more, we conclude the improvement strategy for next generation of oncolytic viruses therapies.

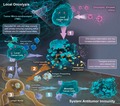

## 1 BACKGROUND

**Figure 1:**
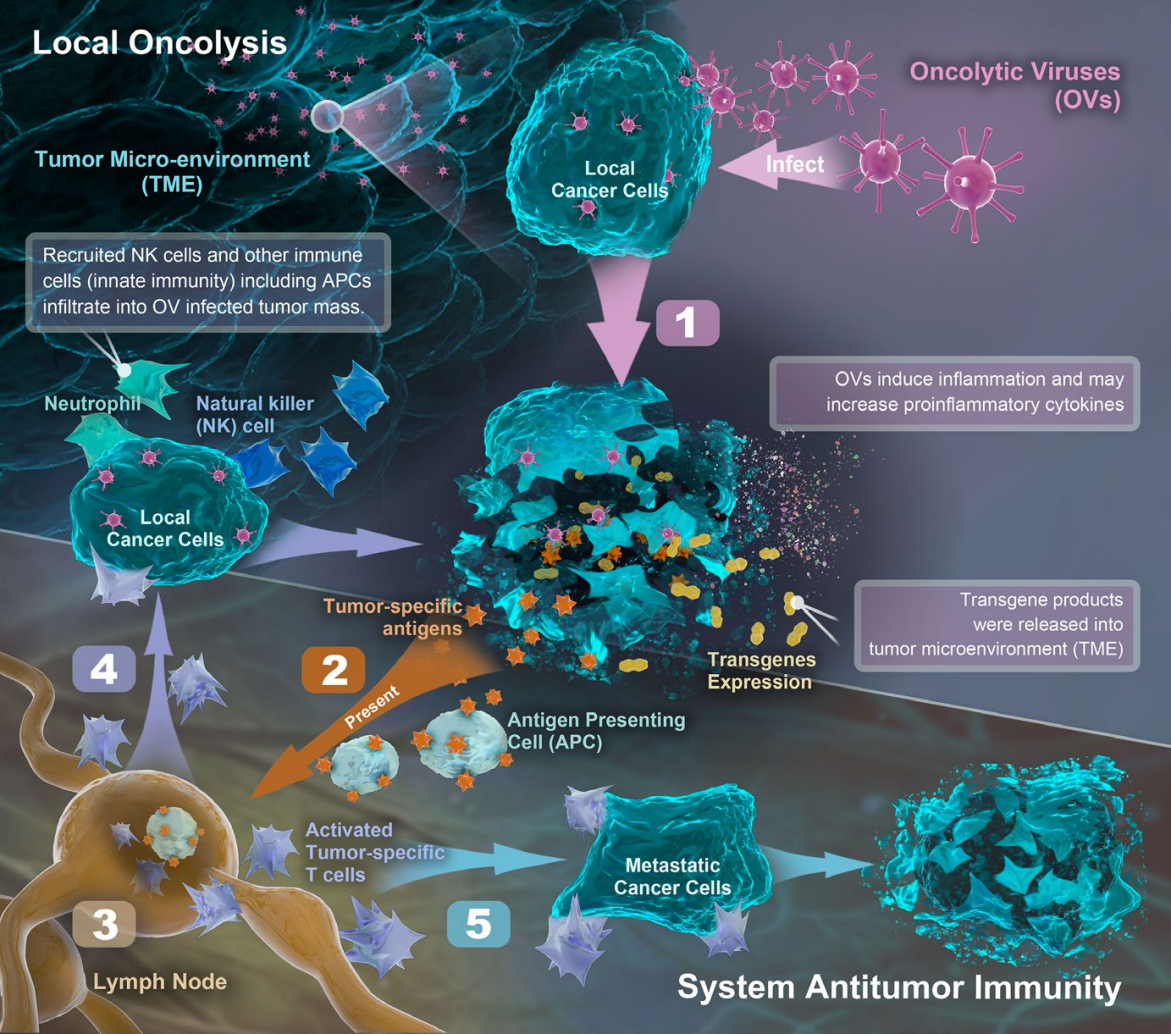
(1) OVs induce immunogenic cell death (ICD). Then oncolysis by OVs causes the release of tumor‐specific antigens (local oncolysis); (2) ~ (3) Tumor‐specific antigens uptake by APCs which migrate to lymph nodes. Antigen‐loaded APCs initiate the activation of tumor‐specific T cells; (4) ~ (5) Tumor‐specific T cells move to local tumor mass (infected) and metastatic cancer cells (uninfected) and exert antitumor effect

Oncolytic virus (OV) selectively replicate in and destroy tumor cells while causing virtually no damage to normal cells. The antitumor effect of oncolytic viruses is primarily achieved in two ways: (a) direct tumor lysis; and (b) induction of an antitumor immune response in animal or humans. On the one hand, because of defective anti‐viral pathways (ie interferon response pathway), tumor cells are unable to stop OV replication and undergo cell lysis and death following infection. The viral progeny are released from the infected tumor cells and spread to uninfected tumor cells, which causes a sustained antineoplastic response.^1^, ^2^ On the other hand, the infection of tumor cells express pathogen‐associated molecular pattern molecules (PAMPs) and damage‐associated molecular pattern molecules (DAMPs), which induces the innate immune response through activation of toll‐like receptors. In turn, the activation of the innate immune system facilitates presentation of viral antigens and tumor‐associated antigens (TAAs) to prime the adaptive immune response.^3^ Of note, several common oncolytic viruses (eg, adenovirus, herpes simplex virus [HSV], vesicular stomatitis virus [VSV], vaccinia virus, and reovirus; Figure 1) have been proven to promote antitumor immunity.^4^, ^5^, ^6^, ^7^, ^8^ Furthermore, oncolytic viruses armed with exogenous immune‐stimulating can exert profound antitumor effects via synergistic stimulation of anti‐cancer immunity.^4^

**Figure 2:**
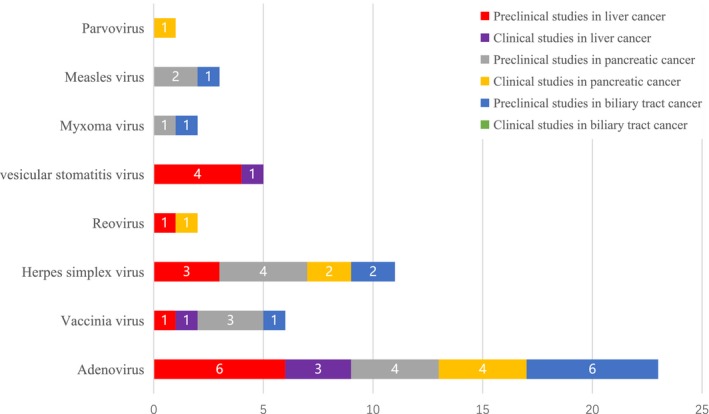
Number of published or registered preclinical and clinical studies for oncolytic virus in hepato‐bilio‐pancreatic cancer Adenovirus is the most widely used. There are few related clinical trials, and most of the existing clinical trials are only in Phase I or Phase II clinical trials

The traditional treatment for hepatocellular carcinoma (HCC), pancreatic cancer, and biliary tract cancer primarily include surgical resection, radiotherapy, and chemotherapy; however, the outcomes of these interventions remain unsatisfactory. Moreover although immunotherapeutic strategies including the PD‐1/CTLA4 antibody and chimeric antigen receptor T cells (CAR T cells) have been proven to have good clinical efficacy in certain tumor types,^9^ the complex immune microenvironment of hepato‐bilio‐pancreatic cancer appears to limit their efficacy.^10^ Given the broad immune‐stimulating and oncolytic activities of OV, it is tempting to speculate that they may be important components, alone and/or in combination with currently available immunotherapies, of successful treatment strategies for hard to treat gastrointestinal (GI) cancers. Here, we systematically review and summarize the current status of treatment and the application of oncolytic viruses for HCC, pancreatic cancer, and biliary tract cancer (Figure 2).

## 2 ONCOLYTIC VIRUSES AS A NOVEL TREATMENT FOR HCC, PANCREATIC CANCER, AND BILIARY TRACT CANCER

### 2.1 Adenoviruses

**Figure 3:**
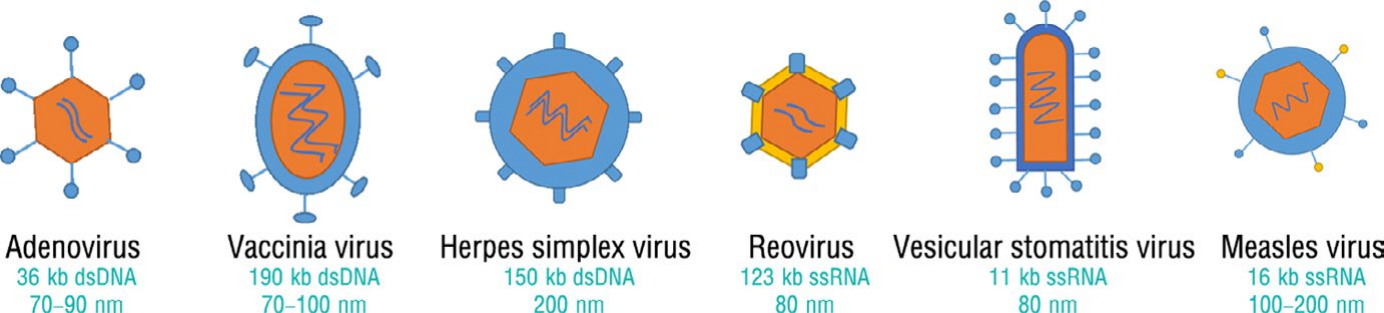
Properties of the oncolytic viruses for hepato‐bilio‐pancreatic cancer and several well‐validated oncolytic viruses are listed. The yellow region represents the capsid and the blue region represents the envelope. Both adenovirus and reovirus are non‐enveloped viruses. The values represent the range of minimum diameter of the capsid. dsDNA, double‐stranded DNA; ssRNA, single‐stranded RNA

Adenoviruses are a non‐enveloped double‐stranded DNA viruses of approximately 36 kb genome that can be divided into 57 different serotypes (Figure 3). Among these serotypes, Ad2 and Ad5, which belong to subgroup C adenoviruses, are the most commonly used adenoviral vectors.^11^, ^12^ Subgroup C adenoviruses infect host cells by binding to the coxsackie adenovirus receptor (CAR) and their transcription requires two key viral genes (E1A and E1B).^13^, ^14^ The protein encoded by E1A orchestrates activation of the E2F transcription factor and initiates the cell cycle of the host cell during the quiescent phase.^13^ The protein encoded by E1B is divided into the E1B‐55 kD protein and E1B‐19 kD protein. The E1B‐55 kD protein binds to p53 and induces its degradation, whereas the E1B‐19 kD protein—similar to the anti‐apoptotic factor Bcl2, prevents the premature apoptosis of infected cells.^11^, ^15^ The advantages of using adenoviruses are their high viral titers, ease of engineering‐in of foreign genes into its moderate size (26‐46 Kbp) DNA genome, and the breadth of clinical experience around their use.^16^ Furthermore, adenoviruses can replicate in host cells at large quantities, and the viral genes cannot integrate into the host cell genome, which improves its safety.^11^

#### 2.1.1 Adenoviruses in liver cancer

Oncolytic adenoviruses can be designed to specifically target liver cancer cells using several different methods. The first method is to specifically target the specific signaling pathways in tumors by altering the key adenovirus replication genes.^12^ This design is primarily achieved by deleting either the E1A or E1B genes. ONYX‐015 is a typical engineered adenovirus (Ad2 and Ad5 hybrid), in which the E1B gene is deleted using gene editing technology to prevent the formation of the E1B‐55 kD protein without affecting the E1B‐19 kD protein, and only replicates in p53‐deficient cells.^17^ In both phase I and phase II clinical trials, intravenous and intratumoral injections of ONYX‐015 were shown to be safe, well tolerated, and exhibited no dose‐limiting toxicity; despite the ubiquity of anti‐adenoviral antibodies before treatment, ONYX‐015 showed some evidence of clinical benefit.^18^, ^19^, ^20^ Further studies have shown that the deletion of the E1B‐55 kD protein played a selective oncolytic role by mediating late viral mRNA nuclear export rather than p53 degradation.^20^ The second method is to control the expression of key genes required for virus replication using a specific promoter of liver cancer tissue (eg, GD55, CNHK500, and SG7011let7T).^21^, ^22^, ^23^, ^24^ ZD55 is an E1B‐deficient Ad5 similar to ONYX‐015.^25^ The AFP‐regulated oncolytic adenovirus (ZD55, in which the endogenous E1A promoter was replaced by the AFP promoter) and GOLPH2‐regulated oncolytic adenovirus, GD55 (ZD55, in which the endogenous E1A promoter was replaced by the GOLPH2 promoter) were designed based on the high expression of AFP and GOLPH2 in HCC, respectively. These OV demonstrated higher specificity against HCC compared to other tumors, as well as a more pronounced antitumor effect, and GD55 induced less damage to normal liver cells compared to ZD55.^23^, ^26^ The third method is to specifically target the liver cancer cell receptor by engineering adenoviral capsids.^27^

To enhance the antitumor effect of oncolytic adenoviruses, a variety of ZD55‐gene systems have been constructed, which preserve tumor targeting and carry different therapeutic genes (eg, ZD55‐Smac/ZD55‐TRAIL and ZD55‐IFN‐β). Tumor necrosis factor‐related apoptosis‐inducing ligand (TRAIL) induces tumor cell apoptosis through the death receptor pathway and various caspases, to which HCC is highly resistant,^28^, ^29^ via in part inhibition of caspase activation by inhibitor of apoptosis proteins (IAP) and the X‐linked inhibitor of apoptosis protein (XIAP).^30^ Thus, ZD55‐Smac/ZD55‐TRAIL also incorporates the mitochondrial‐derived activator of caspases (Smac) which abolishes IAP inhibition of caspases and enhances tumor cell sensitivity to the TRAIL pathway.^29^ The combination of ZD55‐Smac and ZD55‐TRAIL proved to effectively reduce the levels of IAP and XIAP expression and promote HCC cell apoptosis in HCC cell lines in vitro and HCC xenografts in vivo, whereas the effect of either ZD55‐Smac or ZD55‐TRAIL alone was significantly attenuated.^29^ However, expressing pro‐apoptosis gene by an OV is never a good idea since once the gene is expressed, the cells infected will die first, which eliminates the virus replication in the cell and also inhibits virus dissemination inside of tumor mass. ZD55‐IFN‐β, a ZD55 expressing IFN‐β, showed significant antitumor effects and the high level of IFN‐β expression in the HCC cell lines, as well as HCC xenografts. In addition, no obvious cytotoxic or apoptotic effects were detected in normal cells infected with ZD55‐IFN‐β, which indicate that the toxic effect of IFN‐β did not impact the cells in normal tissues.^31^

#### 2.1.2 Adenoviruses in pancreatic cancer

ONYX‐015 has been demonstrated to induce tumor‐specific cytolysis and antitumor efficacy in both pancreatic cancer cell lines in vitro and mice xenografts in vivo.^32^ Moreover the efficacy was significantly enhanced when combined with chemotherapy, including cisplatin and 5‐fluorouracil.^32^ However, a clinical trial showed limited clinical efficacy of the intratumoral endoscopic ultrasound injection of ONYX‐015 combined with an intravenous injection of gemcitabine for unresectable pancreatic cancer.^33^ This may be because pancreatic tumors are highly fibrotic with a high ratio of normal cells to tumor cells, which may highly limit virus dissemination in the tumor since, by definition, OVs cannot replicate in nontumor cells. Therefore, it is plausible that oncolytic virus should be designed to enhance viral spread and breakdown the extracellular matrix of pancreatic tumors.^34^

Several novel oncolytic adenoviruses currently being researched exhibit exciting antitumor activity against pancreatic cancer in preclinical studies. For example, Ad5ΔE1B19K, the adenoviral mutants with an anti‐apoptotic E1B‐19 kD gene deletion, could selectively kill pancreatic cancer cells in vitro and xenografts in vivo when combined with gemcitabine.^35^ A similar oncolytic adenovirus (AdΔΔ) with the deletions in both the *E1ACR2* (another anti‐apoptotic gene) and *E1B19K* genes displayed enhanced antitumor efficacy combined with the chemotherapeutics, docetaxel and mitoxantrone, in pancreatic cancer xenografts in vivo and decreased levels of virus in normal cells compared to the single‐deleted AdΔCR2 mutant.^36^ Telomelysin (OBP‐401), in which the hTERT promoter controls viral replication with a fully functional viral E3 region, only replicates in cells with upregulated hTERT (eg, pancreatic cancer cells).^37^ Telomelysin effectively lysed pancreatic cancer cells and shrank xenografted tumors alone or in combination with docetaxel.^38^ In CRAd‐Cans, deleting the E1B‐55 kD gene and carrying *canstatin* (an angiogenesis inhibitor gene), showed the synergistic effects of antitumor therapy and anti‐angiogenesis therapy.^39^

Most pancreatic cancers demonstrate pRb hyperphosphorylation,^40^ and two oncolytic adenoviruses, LoAd703 and VCN‐01, have been constructed to replicate in pancreatic cancer cells with a disrupted Rb pathway and several related clinical trials are currently underway. LoAd703 is an Ad5/35 expressing human CD40L and 4‐1BBL. CD40L can induce the adaptive immune response and CD40‐mediated tumor cell apoptosis, whereas 4‐1BBL enhances immunological memory and expands T and NK cells, modulating the pancreatic cancer stroma to support the antitumor response.^41^ LoAd703 can efficiently lyse pancreatic cancer cell lines in vitro and reduce the established tumors in xenograft models in vivo.^41^ A Phase I/II clinical trial is currently underway to actively recruit patients to assess the efficacy and safety of an intratumoral injection of LOAd703 combined with gemcitabine and nab‐paclitaxel as a treatment for pancreatic cancer. Another adenovirus (VCN‐01) has been engineered to express hyaluronidase and RGD‐modified fibers, which improves the half‐life of viruses in the blood and enhances intratumoral spread, resulting in antitumor activity in mice and Syrian hamster pancreatic cancer xenografts models after intravenous or intratumoral injection.^42^ A Phase I dose escalation trial of VCN‐01 by intratumoral injection combined with gemcitabine and abraxane determining the safety and tolerability in patients with advanced pancreatic cancer has been completed; however, the results have yet to be reported (NCT02045589).

#### 2.1.3 Adenoviruses in biliary tract cancer

Previous reports have revealed that an adenovirus expressing p27KIP1 induces apoptosis against cholangiocarcinoma cells by triggering extracellular Fas ligand expression.^43^ To further enhance the tumor specificity and viral infectivity of the adenovirus‐based therapies in cholangiocarcinoma cells, Zhu et al designed several novel recombinant adenoviruses with a survivin promoter and capsid modifications. The survivin promoter has extremely low activity in normal human tissues, especially in the liver, but exhibited higher activity in cholangiocarcinoma cells, indicating decreased toxicity to the human liver and higher tumor specificity. Moreover, three different capsid modifications (RGD, F5/3, and pk7) enhanced the levels of viral infectivity; however, the replication ratios of these adenoviruses are significantly lower than the wild type virus, suggesting that the capsid modification enhanced viral infectivity and impaired viral replication.^44^

AxdAdB‐3, a double‐restricted Ad with a mutant E1A and E1B‐55kD deletion, showed effective and selective replication and oncolysis of gallbladder cancer (GBC) cells in vitro and in vivo with reduced negative effects on normal cells.^45^ Moreover AxdAdB‐3‐F/RGD, a novel AxdAdB‐3 with RGD‐modified fibers via the incorporation of an Arg‐Gly‐Asp (RGD) motif into the HI‐loop of Ad5 fiber‐knob region, can enhance infectivity by increasing viral interaction with the integrins that are abundantly expressed in almost all biliary cancer cells.^46^, ^47^ AxdAdB3‐F/RGD exhibited efficient replication and potent oncolysis in both CAR‐positive and CAR‐negative biliary cancer cells and caused a marked inhibition of tumor growth in mouse xenografts, whereas AxdAdB3 only infected biliary cancer cells with preserved CAR expression.^46^ In another approach, the use of a chimeric construct with the shaft from an Ad5 serotype and the knob from Ad3 serotype enhanced transduction in several CAR‐negative cancer cells.^47^, ^48^

### 2.2 Vaccinia virus

Vaccinia virus is a double‐stranded DNA virus that replicates and spreads rapidly (Figure 3). Importantly, vaccinia virus harbors the thymidine kinase (TK) gene, which encodes the TK, an enzyme necessary for viral replication that is ubiquitously expressed in malignant cancer cells but rarely expressed in normal cells.^49^ Thus, by removing the TK gene, vaccinia virus can only target malignant cancer cells, since the defective vaccinia virus can only replicate using the TK of host cells.^50^ Vaccinia virus can secrete viral proteins to activate host cell EGFR‐RAS pathway to further synthesize TK, which improves the efficiency of virus infection and promotes the destruction of tumor cells.^51^ The disabled interferon response of malignant tumor cells is also one of the factors driving vaccinia virus to target malignant tumor cells.^52^ Moreover vaccinia virus exhibits effectiveness and stability when given systemically and is resistant to the effects of antibodies and complement.^53^ The intravenous injection of vaccinia virus has been shown to be effective in tumor xenografts, despite the presence of high antibody titers.^54^

#### 2.2.1 Vaccinia virus in liver cancer

The vaccinia virus currently used in clinical liver cancer research primarily involves JX‐594. JX‐594 is a derivative of the vaccinia virus Wyeth strain (a smallpox vaccine), which was widely used in global vaccination programs to eradicate small pox due to its proven safety in humans.^55^ The use of a vaccinia virus with a single knockout of the TK gene (CVV) effectively removed liver cancer cells in animal models.^56^ JX‐594 contains two genes that were inserted into the TK gene region: (a) a gene encoding hGM‐CSF, which promotes myeloid and dendritic cell maturation to induce antitumor immunity and inhibit tumor blood vessels, but may stimulate myeloid‐derived suppressor cells (MDSCs) resulting in diminished innate and adaptive antitumor responses in numerous cancers^57^, ^58^, ^59^; and (b) *lac‐Z*, which encodes β‐galactosidase as a surrogate marker to assess viral replication.^55^ Current clinical trials have demonstrated that JX‐594 is safe and well‐tolerated when used to treat liver cancer patients and that intriguingly there is no correlation between serum antibody levels and JX‐594 replication, safety, and antitumor activity.^60^, ^61^, ^62^ Kim et al confirmed that JX‐594 was well‐tolerated in rabbits and rat liver cancer models with a significant antitumor effect.^63^ Park et al found that in patients with refractory primary liver cancer and metastatic liver cancer, JX‐594 could be detected in both injected and uninjected tumors, and exhibited antitumor effects.^53^ Grade I‐III flu‐like symptoms were common adverse reactions following an intratumoral injection of JX‐594, and several patients displayed transient grade I‐III dose‐related thrombocytopenia while Grade III hyperbilirubinemia was dose‐limiting. In addition, the maximum tolerated dose was 1 × 10^9^ pfu.^53^ Subsequent studies have not found dose‐limiting toxicities for JX‐594, and suggested the maximum feasible dose is 2 × 10^9^ pfu.^60^ Moreover, Heo et al demonstrated systemic and long‐term efficacy of a direct intratumoral injection of JX‐594 in patients with advanced HCC and the dose was significantly associated with overall survival; the overall survival for the high dose (1 × 10^9^ pfu) treatment was approximately twice that of the low dose group (1 × 10^8^ pfu) (14.1 m vs 6.7 m) with comparable safety.^61^ A phase II clinical trial of advanced liver cancer further confirmed that the dose of a direct intratumoral injection of JX‐594 was significantly associated with the overall survival.^64^ In addition, Cripe et al evaluated the safety of an intratumoral injection of JX‐594 in children with liver cancer, and found that an intratumoral injection of JX‐594 was safe and the side effects were primarily flu‐like symptoms and skin pustules.^62^ Since patients with liver cancer are often associated with cirrhosis and viral hepatitis, traditional treatments do not induce an antiviral response; however, JX‐594 induces high concentrations of antiviral cytokines that inhibit HBV, which may be effective for treating HBV‐related HCC.^65^ Compared with traditional sorafenib treatment, JX‐594 is also associated with a longer overall survival and fewer adverse reactions; however, additional clinical trials are needed to further verify the safety of JX‐594 and explore the possibilities of combining JX‐594 with other therapies. A phase III clinical trial (PHOCUS) investigating the combination of JX‐594 and sorafenib for the treatment of advanced liver cancer is currently underway (NCT02562755).

#### 2.2.2 Vaccinia virus in pancreatic cancer

GLV‐1h68 (GL‐ONC1) is a replication‐competent virus containing the marker genes *ruc‐gfp* and mutations in the F14.5L, J2R, and A56R loci, which disrupts TK and hemagglutinin. GLV‐1h68 has demonstrated safety and antitumor efficacy in PANC‐1 cell lines in vitro and PANC‐1 pancreatic tumor xenografts via systemic injection in vivo.^66^ Moreover GLV‐1h68 combined with cisplatin or gemcitabine resulted in enhanced therapeutic efficacy.^66^ To enhance the role of the virus as a cancer treatment, multiple therapeutic genes have been engineered for expression in vaccinia virus. The Lister vaccinia virus vaccine strain was designed to encode the endostatin‐angiostatin fusion gene (VVhEA), and showed significant potent antitumor activity against human pancreatic tumor cells in vitro, a high selectivity for cancer cells, and inhibition of angiogenesis and tumor regression of human pancreatic tumor xenograft tumors in mice following both intravenous and intratumoral injection.^67^ It is important to note that VVhEA was effective against pancreatic tumors insensitive to oncolytic adenoviruses.^67^ Another Lister vaccine strain (VV‐IL‐10) that encoded interleukin (IL)‐10 and lacked TK has demonstrated superior antitumor efficacy in mice with subcutaneous pancreatic cancer that was also associated with long‐term antitumor immunity.^68^ Although, no significant difference was observed in in vitro antitumor activity when VV‐IL‐10 was compared with the control VV, the presence of IL‐10 which prevents host immune inhibition of viral replication, resulted in greater therapeutic efficacy in vivo of VV‐IL‐10.^68^, ^69^

#### 2.2.3 Vaccinia virus in biliary tract cancer

GLV‐1h68 has also demonstrated oncolytic activity against cholangiocarcinoma cell lines in vitro*,* and was well tolerated and exhibited antitumor efficacy in xenograft bearing athymic nude mice.^70^ A phase I clinical trial is currently evaluating the safety and efficacy of GLV‐1h68, which is delivered as a bolus intravenous injection, in patients with solid organ cancers, including cholangiocarcinoma (NCT02714374).

### 2.3 Herpes simplex virus

Herpes simplex virus (HSV) is a double‐stranded DNA virus that can be divided into HSV‐1 and HSV‐2 according to the specific serotype (Figure 3). Currently, HSV‐1 is primarily used in clinical oncolytic therapy, whereas there are some studies which show that HSV‐2 is also effective against a variety of tumors; interestingly, a tumor cell subpopulation that only responds to treatment with HSV‐2 has been found.^71^, ^72^ Compared with other viruses, HSV can infect and quickly kill tumor cells, supporting rapid viral replication and spread of the virus within a tumor mass. Moreover the safety of HSV can be ensured through the use of anti‐HSV drugs (eg, acyclovir).^71^

#### 2.3.1 HSV in liver cancer

HSV‐1 is primarily used for the treatment of liver cancer. The genome of HSV‐1 is approximately 150 kb, including many non‐essential genes that have no significant effect on viral replication and can be modified without losing the oncolytic effect of the virus.^73^ In the HSV‐1 OV, LCSOV, expression of the viral glycoprotein H gene is driven by the liver‐specific apolipoprotein E (apoE)‐AAT promoter and contains additional miRNA complementary sequences of miR‐122a, miR‐124a, and let‐7 inserted into the same 3′ UTR region of the modified *gH* gene.^74^ miR‐122a is a liver‐specific miRNA that is expressed at low levels only in HCC, whereas miR‐124a and let‐7 are down‐regulated or lacking in malignant cancer cells enabling translational control of gH expression in LCSOV infected normal cells.^74^, ^75^ LCSOV also displays high selectivity and effective killing in HCC xenografts and cell lines, and significantly reduces tumor volume with minimal toxicity.^74^ G47Δ is a third‐generation oncolytic HSV with mutations in the *γ34.5, ICP6, and ICP47* genes. The *γ34.5* gene production can prevent the host cell from stopping translation due to viral infection, allowing viral proteins to be continuously synthesized. Since tumor cells often lack control over translation and the antiviral response, HSV with the *γ34.5* gene mutation can replicate in tumor cells.^73^, ^76^ The *ICP6* gene product is required for the replication of viruses in noncycling cells, so mutations in the *ICP6* gene enable HSV to selectively replicate in constantly dividing cells (eg, tumor cells).^77^ Moreover, an *ICP47* gene mutation can amplify the targeting of tumor cells caused by a *γ34.5* gene mutation and stimulate T cells to enhance the immune response and antitumor immunity to virus‐infected tumor cells.^73^, ^78^ G47Δ requires only a very low MOI to effectively kill a variety of different HCC cell lines and inhibit tumor growth in HCC xenografts with no significant toxicity.^78^ HSV‐1‐T‐01 is similar to G47Δ, in which the *γ34.5* and *ICP6* genes were deleted and the *ICP6* gene was replaced with the *LacZ* gene. HSV‐1‐T‐01 was found to inhibit tumor growth in both HCC cell lines and xenografts, and significantly improved the antitumor effect by enhancing T cell‐mediated immunity.^79^

#### 2.3.2 HSV in pancreatic cancer

Several preclinical studies have demonstrated the antitumor activity of both HSV‐1 and HSV‐2 for pancreatic cancer. G207 is a typical engineered HSV‐1 with deletions in both copies of *γ34.5* and genetic inactivation of ICP6, which replicated in and lysed human pancreatic cancer cells in vitro.^80^ Another engineered HSV‐1, NV1020, had a deletion in only one copy of *γ34.5* also displayed effective viral replication and cell lysis in human pancreatic cancer cells in vitro*,* as well as a higher production of viral progeny.^81^ Both the injection of G207 and NV1020 into athymic mice xenografts induced complete pancreatic tumor eradication in 25% and 40% of mice, respectively.^81^ FusOn‐H2 is an HSV‐2 construct with a deletion in the PK domain which encodes serine/threonine protein kinase activity and replicates in cells with an activated Ras signaling pathway. FusOn‐H2 could eradicate subcutaneous tumors and orthotopic tumors in human pancreatic cancer xenografts following an intratumor and systemic injection, respectively.^82^

A Phase I clinical trial evaluated the efficacy of HF10, a naturally mutated HSV‐1, in six patients with unresectable pancreatic cancer. No adverse effects were observed and HF10 infection stimulated the host antitumor immune responses and increased the number of NK cells, CD4+ cells, CD8+ cells, and macrophages after virus injection; four of the six patients showed clinical efficacy with longer survival.^83^ Additionally, talimogene laherparepvec (T‐VEC), a modified HSV‐1 with deletions in γ34.5 and ICP47, and also expressing GM‐CSF, has been approved by the FDA as the first oncolytic virus therapy for the treatment of melanoma. Unfortunately, a Phase I trial assessing the safety of T‐VEC in 17 patients with unresectable pancreatic cancer showed limited efficacy and various adverse events and only two patients completed the trial (NCT00402025).

#### 2.3.3 HSV in biliary tract cancer

The study of oncolytic therapy for cholangiocarcinoma remains in the preclinical stage. NV1203 is an attenuated HSV with a UL56 deletion, a single copy of ICP0, ICP4, and γ34.5, as well as the insertion of the *Escherichia coli lacZ* marker gene into the ICP47 locus. UL56 is associated with the pathogenicity and neuroinvasiveness of HSV, and the lack of UL56 attenuates the virus.^84^ In addition, ICP0 is associated with both lytic and latent HSV infections, and ICP0‐null HSV‐1 is extremely sensitive to IFN and PML‐mediated disruption of the viral lifecycle; however, since IFN and PML are downregulated in most tumors, this virus can specifically target cancer cells.^73^, ^85^, ^86^ Combination treatment with low‐dose external radiation therapy (XRT) and NV1203 was highly tumoricidal, both in vitro and in vivo,^87^ though the greatest tumor volume decrease was observed in the YoMi cell line, which correlated with upregulation of growth arrest and DNA damage protein 34 (GADD34) by XRT.^87^ GADD34 has significant homology to γ34.5 and improves the specificity of the virus for targeting tumor cells.^87^

G207 is a HSV‐1 mutant with deletions in both the *γ34.5* and *ICP6* genes, and its safety has been demonstrated in humans.^88^ Nearly total cell killing was observed in five gallbladder carcinoma cell lines within 72 hours of in vitro G207 infection.^89^ Moreover an intratumor injection of G207 (1 × 10^7^ pfu) in immunocompetent hamsters bearing established subcutaneous KIGB‐5 tumors displayed a significant inhibition of tumor growth and prolonged survival.^89^ Interestingly, a decreased antitumor effect was observed in athymic mice bearing KIGB‐5 tumors, suggesting that the systemic antitumor effect of G207 is partly mediated by T cells.^89^

### 2.4 Reovirus

Reovirus is a double‐stranded RNA virus that is ubiquitous in nature, found in untreated sewage, stagnant water, and rivers worldwide (Figure 3). In normal cells, a reovirus infection activates the PKR pathway of the host cell. PKR is a serine/threonine protein kinase involved in antiviral host defense, functioning to inhibit reovirus translation, replication, and further infection.^90^ In cells in which the Ras signaling pathway is activated, the PKR pathway is inhibited, resulting in viral replication and eventually destroying the host cells. Ras gene mutations have been found in various human tumors, including pancreatic, colon, and lung cancer, suggesting the potential of using reoviruses to treat tumors.^91^, ^92^ Ras transformation mediates reovirus oncolysis for cells exhibiting activated Ras signaling by enhancing viral uncoating, particle infectivity, and apoptosis‐dependent release of progeny virions.^93^ Reovirus also promotes direct oncolytic effects and the antitumor immune response by altering the tumor microenvironment due to the substantial secretion of inflammatory cytokines and chemokines by NK cells, DCs, and cytotoxic T cells.^94^ Moreover an intravenous reovirus injection is safe, displays no dose‐limiting toxicity, and has potent antitumor effects.^95^ Currently, although the reovirus type 3 Dearing strain is most commonly used in clinical studies, there are only a few studies using reovirus to treat liver and biliary tract cancer.

#### 2.4.1 Reovirus in liver cancer

Most liver cancers are associated with oncogenic viral infections (eg, HBV [54%] and HCV [31%]).^96^ Park et al found that the oncogenic protein, HBX, produced by HBV inhibited the oncolysis of reovirus in HCC; thus, reovirus had a limited therapeutic effect on HBV‐HCC.^96^ In contrast, Samson et al demonstrated that reovirus could effectively inhibit HCV replication in HCV‐HCC mouse models and cell lines, demonstrating significant anti‐HCC responses through inducing innate immune activation and IFN production.^97^

#### 2.4.2 Reovirus in pancreatic cancer

Approximately 90% of pancreatic cancers have *K‐ras* gene mutations; thus, reoviruses are oncolytic toward cancer cells displaying activated Ras signaling.^92^ Reovirus (serotype 3) has shown significant antitumor effects in four human pancreatic cancer cell lines with K‐ras mutations and BxPC3 with a normal Ras proto‐oncogene, and was also an effective therapy in mice with Panc1 and BxPC3 xenografts mice in vivo.^98^ An intraportal administration of reovirus decreased the number and size of liver metastases from pancreatic cancer without any toxicity to normal hepatic tissue in hamsters.^99^ Furthermore, an intraperitoneal administration of reovirus effectively controlled the peritoneal dissemination of pancreatic cancer in hamsters.^100^

Pelareorep (REOLYSIN^®^) is a proprietary isolate of the human reovirus type 3 Dearing strain and demonstrates a potential anticancer effect towards several cancers when used as a mono and/or combination therapy.^101^ A phase II study of pelareorep combined with gemcitabine therapy was evaluated in 34 patients with advanced pancreatic adenocarcinoma. The combined therapy was well tolerated without serious adverse events and the median progression‐free survival (PFS) was 3.4 months (median overall survival [OS]: 10.2 months) with a 1‐ and 2‐year survival rate of 45% and 24%, respectively, which were the highest observed rates in the similar studies, which demonstrated that pelareorep can complement gemcitabine treatment in pancreatic cancer.^102^ However, another phase II study of pelareorep combined with paclitaxel/carboplatin therapy compared with paclitaxel/carboplatin therapy alone was evaluated in 73 patients with metastatic pancreatic adenocarcinoma; no significant differences were found in the response rate, PFS, or OS between the two therapies due to insurmountable immune suppression and elevated expression of CTLA4 on T cells may be the key factor limiting the oncolytic efficacy in patients with pancreatic cancer.^103^

### 2.5 Vesicular stomatitis virus

Vesicular stomatitis virus (VSV) is a negative‐stranded RNA virus that specifically targets tumor cells due to the reduced ability of tumor cells to resist VSV infection (Figure 3). An engineered VSV that expresses β‐gal (rVSV‐β‐gal) demonstrated effective viral transduction, tumor‐selective viral replication, and extensive oncolytic effects in HCC cells, and prolonged survival of Buffalo rats bearing orthotopic HCC without significant cytotoxicity or liver damage. Further experiments showed that while the survival time of rats treated with rVSV‐β‐gal was significantly prolonged, viral spread between solid tumor cells was limited.^104^ To overcome this problem, recombinant virus VSV‐NDV was constructed to induce the formation of syncytia between tumor cells to promote efficient viral spread. The VSV membrane surface glycoprotein is replaced by Newcastle disease virus (NDV) hemagglutinin‐neuraminidase (HN) protein and modified fusion (F) membrane protein, which retain the ability for rapid replication while increasing the safety and the ability of virions to spread between cells and enhance the oncolytic effect.^105^ VSV‐NDV‐induced extensive syncytia formation and enhanced tumor cytotoxicity were observed in both in vivo and in vitro HCC models without inducing significant peripheral hepatic parenchymal damage.^106^ VSV (MΔ51)‐M3 is a recombinant VSV with amino acid 51 deleted from the VSV‐M protein and also expresses M3, a chemokine‐binding protein from murine gammaherpesvirus‐68. The deletion of amino acid 51 from the VSV‐M protein results in the loss of the ability of the virus to inhibit mRNA transport in host cells; therefore, IFN and cytokine expression is increased in infected cells, which increases the safety of rVSV but induces a stronger inflammatory response and reduces the oncolytic effect of rVSV.^107^ The addition of M3 antagonizes chemokine signaling and reduces immune infiltration allowing survival of the virus and oncolysis to continue. In an HCC‐bearing mouse model of hepatic artery perfusion, treatment with rVSV (MΔ51)‐M3 decreased infiltration of neutrophils and NK cells in the lesion, while the viral titer increased, the oncolytic effect was enhanced, and more importantly, no obvious systemic and organ toxicities were observed.^107^

### 2.6 Myxoma virus

Myxoma virus (MYXV) is a member of the poxvirus family that has a double‐stranded DNA genome and is pathogenic to rabbits, but not humans.^108^ In addition, a MYXV infection may be prevented through protective interferon responses induced in species other that rabbits, which results in a narrow host tropism.^109^ However, MYXV can replicate in cells with an activated Akt pathway, as well as p53 or Rb dysfunction.^110^, ^111^ Akt is a serine/threonine kinase that regulates cellular proliferation and death but is upregulated in several human cancer cells.^112^ Therefore, MYXV can be used to selectively target many cancer cells and has been shown to be effective at infecting and killing 70% of tested tumor cell lines.^113^ Moreover MYXV can productively infect, replicate in, and lyse human pancreatic cancer cells in vitro and prolong survival in mouse xenografts in vivo*.* 114 In addition, the combined use of MYXV and gemcitabine displays a robust antitumor effect.^115^

The effective tumor cell killing ability of MYXV has been shown in a variety of human gallbladder carcinoma cell lines.^116^ Both rapamycin and hyaluronan can effectively enhance the oncolytic ability of MYXV in vitro, but only hyaluronan can enhance the antitumor effects of MYXV in vivo and prolong the survival of GBC tumor‐bearing mice *via* the interaction between HA and CD44 which results in increased Akt signaling.^117^ There are no related clinical trials in human subjects are currently ongoing.

### 2.7 Measles virus

Measles virus (MV) is a negative‐stranded RNA paramyxovirus (Figure 3) and the vaccine strains of the virus widely used for measles prevention worldwide have demonstrated excellent safety.^118^ MV enters cells through the CD46 receptor, a membrane‐associated protein that protects cells against complement‐mediated lysis that is overexpressed in tumors but exhibits low levels of expression in normal cells; thus, MV preferentially infects tumor cells.^119^, ^120^ The virus kills tumor cells via cell‐to‐cell fusion and the formation of mononuclear cell aggregates.^121^ MV strains have demonstrated potent antitumor activity in multiple tumor models, including both solid tumors and hematologic malignancies.^119^

A MV expressing the sodium iodide symporter reporter gene (MV‐NIS) was found to efficiently infect human pancreatic tumor xenografts in athymic nude mice and facilitated diagnostic imaging of infection.^122^

MeV‐SCD is a measles vaccine virus that has been engineered to express super cytosine deaminase (SCD), a fusion protein consisting of yeast cytosine deaminase and uracil phosphoribosyl transferase.^123^ Since 5‐FU is commonly used for the treatment of carcinomas with low effectiveness in bile duct cancer, SCD can convert the prodrug 5‐fluorocytosine (5‐FC) to 5‐fluorouracil (5‐FU) and subsequently to 5‐fluorouridine‐monophosphate, which inhibits both DNA and protein synthesis.^120^, ^123^, ^124^ MeV‐SCD combined with the administration of 5‐FC displays significant oncolytic ability against cholangiocarcinoma in vitro. In vivo, the intratumoral administration of MeV‐SCD significantly reduced the tumor size and was associated with a significant survival benefit.^123^

### 2.8 Application status of OV therapy in hepato‐bilio‐pancreatic cancer

Tables 1, 2, 3 show the application situation of oncolytic virus in HCC, pancreatic cancer, and biliary tract cancer. Different OV have their unique advantages and disadvantages. For example, As the most widely used virus for research, adenovirus has broad tropism for infecting many human tissues and is conducive to clinical applications.^12^ However, the small genome of adenovirus only allows insertion of small portions of genetic material (not exceeding 8 kb), limiting its ability to deliver multiple antitumor or immune‐stimulating payloads. In contrast, HSV‐1 has a large genome with many genes not necessary for virus replication, thus allowing researchers to manipulate the genome to enhance the oncolytic activity without destroying the ability of virus replication.^73^ Importantly, HSV‐1 is highly immunogenic, directly stimulating NK cells and synergizing with IL‐15 to promote antitumor immunity.^125^, ^126^ However, HSV‐1 spreads from cell to cell, suggesting that intratumoral injection may be the best meansfor delivery while intravenous administration may be not suitable due to multiple physical (ie general “stickiness” to endothelium and blood components) and immunological barriers (inactivation by neutrophils, monocytes, neutralizing antibodies, etc).^127^ Vaccinia virus also has a large genome to accommodate multiple foreign genes and has high transduction efficiency.^128^ But different from HSV which is a neurovirulent human pathogen, the safety of vaccinia virus has been widely demonstrated through its widespread use as a vaccine to eradicate small pox. Most genetically engineered oncolytic viruses have an attenuated viral backbone improving the safety of the virus. However, the oncolysis is correspondingly weakened, so improvements are based on constructing multi‐regulated viral backbones to further increase tumor selectivity, and/or equipping the virus with co‐stimulatory factors to potentiate antitumor immunity. Different from the genetically engineered oncolytic viruses, Reolysin which has oncolytic activity and insufficient space to insert foreign genes, may necessitate combination strategies for maximal therapeutic efficacy. Almost all the combination of Reolysin were with chemotherapy, though the efficacy was not satisfactory.^103^, ^129^ Therefore, current efforts are aimed at combining Reolysin with immunotherapy.^102^ A common strategy is to arm virus with exogenous gene expressing cytokines such as GM‐CSF and IL‐12 to enhance antitumor immunity. It is worth noting that although GM‐CSF has been widely used as a payload in OV, it may lead to suppression of immune responses by activating MDSCs.^57^, ^58^, ^59^ Published preclinical studies have confirmed the effective oncolysis of oncolytic viruses in hepato‐bilio‐pancreatic cancer models both in vitro and in vivo. However, the viruses which are significantly effective in preclinical studies may not be effective in the clinical setting because of many factors within the tumor microenvironment (TME) of hard to treat GI tumors including (a) the biophysical barriers of desmoplasia, high interstitial pressures and hypoxia, and (b) tumor‐derived immunosuppressive factors such as cytokines, chemokines, and suppressor cells.^128^, ^129^

**Table 1:** Application situation of oncolytic virus in HCC

Viral type	Name	Mode of administration	Key features	Study types	Ref./Clinical trail
Adenovirus	ONYX‐015	Intratumoral or intravenous	Disruption of the coding sequence of the E1B‐55kD protein	Phase II	[18]
	CNHK500	Intratumoral or intravenous	The expression of E1A gene is regulated by hTERT promoter and the expression of E1B gene is regulated by hypoxia promoter	Preclinical	[22]
	GD55	Intratumoral	E1B‐55kD protein‐deficient and the endogenous E1A promoter was replaced by the GOLPH2 promoter	Preclinical	[23]
	AD	Intratumoral	E1B‐55kD protein‐deficient and the endogenous E1A promoter was replaced by the AFP promoter	Preclinical	[26]
	ZD55‐Smac/ZD55‐TRAIL	Intratumoral	E1B‐55kD protein‐deficient and arm with Smac and TRAIL genes	Preclinical	[29]
	ZD55‐IFN‐β	Intratumoral	E1B‐55kD protein‐deficient and arm with IFN‐β gene	Preclinical	[31]
Vaccinia virus	CVV	Intratumoral	Deletion of TK gene	Preclinical	[56]
	JX‐594	Intratumoral or intravenous	Deletion of TK gene and insert genes encoding hGM‐CSF and β‐galactosidase	Phase II/III	[60, 63, 64]
HSV‐1	LCSOV	Intratumoral	Additional miRNA complementary sequences of miR‐122a, miR‐124a and let‐7 inserting into the same 3′ UTR region of the modified *gH* gene And viral glycoprotein H gene is linked to liver‐specific apolipoprotein E (apoE)‐AAT promoter	Preclinical	[74]
	G47Δ	Intratumoral	The mutations of *γ34.5, ICP6 and ICP47* gene	Preclinical	[78]
	HSV‐1‐T‐01	Intratumoral	The *ICP*47 and *γ*34.5 loci are deleted and the LacZ gene replaces the ICP6 gene	Preclinical	[79]
VSV	rVSV‐GFP	Intratumoral	Arm with the gene expressing GFP	Preclinical	[130]
	rVSV‐β‐gal	Hepatic artery perfusion	Arm with the gene expressing β‐galactosidase	Preclinical	[104]
	VSV‐NDV	Hepatic artery perfusion	The membrane surface glycoprotein of VSV is replaced by Newcastle disease virus (NDV) hemagglutinin‐neuraminidase (HN) protein and modified fusion (F) membrane protein	Preclinical	[106]
	rVSV(MΔ51)‐M3	Hepatic artery perfusion	Deletes amino acid 51 of the VSV‐M protein and expresses M3	Preclinical	[107]

**Table 2:** Application situation of oncolytic virus in pancreatic cancer

Viral type	Name	Mode of administration	Combination therapy	Key features	Study types	Ref./Clinical trail
Adenovirus	ONYX‐015	Intratumoral	Gemcitabine	Disruption of the coding sequence of the E1B‐55kD protein	Phase II	[33]
	Ad5ΔE1B19K	Intratumoral	Gemcitabine	The expression of E1A gene is regulated by hTERT promoter and the expression of E1B gene is regulated by hypoxia promoter	Preclinical	[35]
	AdΔΔ	Intratumoral	Docetaxel + Mitoxantrone	E1B‐55kD protein‐deficient and the endogenous E1A promoter was replaced by the GOLPH2 promoter	Preclinical	[36]
	OBP‐401	Intratumoral	Docetaxel	E1B‐55kD protein‐deficient and the endogenous E1A promoter was replaced by the AFP promoter	Preclinical	[37]
	CRAd‐Cans	Intratumoral	—	E1B‐55kD protein‐deficient and arm with *canstatin* gene	Preclinical	[39]
	LoAd703	Intratumoral	Gemcitabine + Nab‐paclitaxel	E1B‐55kD protein‐deficient and arm with IFN‐β gene	Phase II	NCT02705196
	VCN‐01	Intratumoral	Gemcitabine + Abraxane	Express hyaluronidase and RGD‐modified fibers	Phase I	NCT02045589/NCT02045602
Vaccinia virus	GLV‐1h68	Intravenous	Cisplatin or Gemcitabine	The LIVP strain with mutations in F14.5L, J2R, and A56R loci	Preclinical	[66]
	VVhEA	Intratumoral or intravenous	—	The Lister vaccine strain armed with the endostatin‐angiostatin fusion gene	Preclinical	[67]
	VV‐IL‐10	Intratumoral	—	The Lister vaccine strain armed with interleukin‐10 and lacking TK	Preclinical	[68, 69]
HSV‐1	G207	Intratumoral	—	Deletions in both copies of γ34.5 and genetic inactivation of ICP6	Preclinical	[80]
	NV1020	Intratumoral	—	Deletion in only one copy of γ34.5	Preclinical	[81]
	HF10	Intratumoral	—	Natural mutation which UL43, 49.5, 55, 56 and latency‐associated transcript are functionally inactivated	Phase I	[83]
	T‐VEC	Intratumoral	—	Deletions in γ34.5 and ICP47 as well as expression of GM‐CSF	Phase I	NCT00402025
HSV‐2	FusOn‐H2	Intratumoral and intravenous	—	Deletion in PK domain	Preclinical	[82]
	L1BR1	Intratumoral	5‐FU + Cisplatin	Anti‐apoptotic gene US3 locus‐deficient	Preclinical	[131]
Reovirus	Pelareorep	Intravenous	Gemcitabine or Paclitaxel + Carboplatin	Unmodified proprietary isolate of reovirus Type 3 Dearing	Phase II	[102, 103]
Myxoma virus	MYXV	Intratumoral	Gemcitabine	Unmodified	Preclinical	[115]
Measles virus	MV‐NIS	Intratumoral	—	Arm with the sodium iodide symporter reporter gene	Preclinical	[122]
	MV‐PNP‐anti‐PSCA	Intratumoral	Gemcitabine	Express the prostate stem cell antigen (PSCA) and the prodrug convertase purine nucleoside phosphorylase (PNP)	Preclinical	[132]
Parvovirus	H‐1PV	Intravenous	—	Unmodified	Phase I/II	NCT02653313

**Table 3:** Application situation of oncolytic virus in biliary tract cancer

Viral type	Name	Mode of administration	Combination therapy	Key features	Cancer types	Study types	Ref./Clinical trail
Adenovirus	AxE1CAUT	Intratumoral	5‐FU and/or Ganciclovir	Deletion of the E1A, E1B, and E3 regions and cDNAs of UPRT and HSV‐tk with the CAG promoter are inserted	Cholangiocarcinoma	Preclinical	[133]
	AxdAdB‐3	Intratumoral	Gene‐directed enzyme prodrug therapy	A mutant E1A and E1B‐55kD deletion	Gallbladder carcinoma	Preclinical	[45]
	AxdAdB‐3‐F/RGD	Intratumoral	—	A mutant E1A and E1B‐55kD deletion and the incorporation of an Arg‐Gly‐Asp motif into the HI‐loop of Ad5 fiberknob region	Gallbladder carcinoma	Preclinical	[46]
	AdSurp‐P53	Intratumoral	—	Survivin promoter‐regulated as well as high expression of p53	Gallbladder carcinoma	Preclinical	[134]
	SG7605‐p53‐11R	Intratumoral	—	Arm with the p53 gene and cell‐penetrating peptide 11R	Gallbladder carcinoma	Preclinical	[135]
Vaccinia virus	GLV‐1h68	Intratumoral	—	The LIVP strain with mutations in F14.5L, J2R, and A56R loci	Cholangiocarcinoma	Preclinical	[70]
HSV‐1	NV1203	Intratumoral	XRT	UL56 deletion as well as a single copy of ICP0, ICP4, γ34.5, and the Escherichia coli lacZ marker gene is inserted into the ICP47 locus	Cholangiocarcinoma	Preclinical	[87]
	G207	Intratumoral	—	Deletions in both copies of γ34.5 and genetic inactivation of ICP6	Gallbladder carcinoma	Preclinical	[89]
Myxoma virus	MYXV	Intratumoral	Rapamycin + Hyaluronan	Unmodified	Gallbladder carcinoma	Preclinical	[116, 117]
Measles virus	MeV‐SCD	Intratumoral	5‐FC	Express super cytosine deaminase	Cholangiocarcinoma	Preclinical	[123]

## 3 CONCLUSION

The current understanding of oncolytic viruses only represents the tip of the iceberg in this field. Due to the limitations of traditional treatments, oncolytic virus therapy currently represents a more promising antitumor therapy, especially for advanced refractory cancers. However, due to the above‐mentioned limitations, we consider that next generation OV therapies will need to maximize immune stimulation, antagonize immunosuppressive cells and soluble factors, and incorporate strategies to eliminate or minimize the biophysical barriers of the TME. Depending on the OV platform, these goals may be accomplished in combination with other therapeutics or by engineering the appropriate genes into the vector.

## CONFLICTS OF INTEREST

The authors declare no conflict of interest.

## AUTHOR CONTRIBUTIONS

Yuwei Li and Yinan Shen contributed equally to this work. All authors contributed to the study conception and design. All authors read and approved the final manuscript.

## Data Availability

Data sharing is not applicable to this article as no new data were created or analyzed in this study.
